# YKL-40 is correlated with FEV_1_ and the asthma control test (ACT) in asthmatic patients: influence of treatment

**DOI:** 10.1186/1471-2466-15-1

**Published:** 2015-01-12

**Authors:** Tianwen Lai, Min Chen, Zaichun Deng, Yingying Lǚ, Dong Wu, Dongming Li, Bin Wu

**Affiliations:** Department of Respiratory and Critical Care Medicine, Institute of Respiratory Diseases, Affiliated Hospital, Guangdong Medicine College, Zhanjiang, 524000 China; Department of Respiratory Medicine, Affiliated Hospital of School of Medicine, Ningbo University, Ningbo, 315020 China

**Keywords:** Asthma, CHI3L1, Exacerbation, YKL-40

## Abstract

**Background:**

YKL-40 is also called chitinase-3-like-1 (CHI3L1) protein and may be a marker for asthma. The aims of the present study were to investigate whether serum YKL-40 levels are stable or decreased in patients with asthma after appropriate treatment and to evaluate the correlation of YKL-40 levels with lung function and asthma control test (ACT) results.

**Methods:**

A total of 103 asthmatic patients (mean age 33.1 ± 0.9 years) with diagnosed asthma were enrolled in our study. All patients underwent a detailed clinical examination and completed the ACT questionnaire, serum YKL-40 measurement, and spirometry before (visit 1) and 8 weeks after initiation of treatment (visit 2).

**Results:**

At the follow-up, the median serum YKL-40 level was significantly decreased compared to the levels at visit 1 (75.2 [55.8-86.8] ng/ml versus 54.5 [46.4-58.4] ng/ml, *p* < 0.001). The serum YKL-40 level was negatively correlated with %FEV_1_ (*r* = -0.37, *p* < 0.001) and ACT score (*r* = -0.26, *p* = 0.007) at visit 1. The change in serum YKL-40 levels between the visits was significantly correlated with changes in FEV_1_ (*r* = -0.28, *p* = 0.006) and ACT score (*r* = -0.22, *p* = 0.037). Patients with elevated YKL-40 levels had significantly greater corticosteroid use than patients with lower levels.

**Conclusions:**

YKL-40 was reduced in the serum of asthmatic patients after appropriate treatment, and the levels correlated with improvements in %FEV_1_ and ACT. High levels of serum YKL-40 may be refractory to current asthma treatments.

**Trial registration:**

ChiCTR-OCC-13003316

**Electronic supplementary material:**

The online version of this article (doi:10.1186/1471-2466-15-1) contains supplementary material, which is available to authorized users.

## Background

Asthma is a common chronic disease characterised by acute inflammation of the airways. Asthma arises from a complex interaction between genetic factors of the evolving immune system in the infant and the environment it encounters [1, 2]. Despite recent guidelines focusing on asthma control, many asthmatic patients remain poorly controlled under specialist care [3]. Patient outcomes could be improved by earlier diagnosis and better monitoring. Therefore, it is critical to identify new biomarkers to measure and monitor inflammation within the lungs of a patient with asthma. The role of chitinase and chitinase-like proteins in inflammation and tissue remodelling in human disease has become an important issue.

The chitinase-like protein YKL-40 is also termed chitinase 3-like 1 (CHI3L1). It is produced at sites of inflammation in many cells and is secreted from macrophages and smooth muscle cells [4]. YKL-40 binds to ubiquitously expressed chitin but lacks chitinase activity. Previous studies have demonstrated that YKL-40 was associated with pathologic conditions characterised by aberrant cell growth, tissue inflammation and remodelling. Examples of pathologic conditions involving YHL-40 include the following: cancers, diabetes, atherosclerosis, rheumatoid arthritis, asthma, chronic obstructive pulmonary disease (COPD), liver fibrosis, idiopathic pulmonary fibrosis and Crohn’s disease [4–17]. The measurement of YKL-40 levels is useful and has diagnostic and prognostic value in several diseases [5, 7, 14].

YKL-40 is synthesised in neutrophil precursors and is stored in the specific granules of neutrophils. YKL-40 secretion is stimulated by the cytokines IL-6, IL-17, and IL-18 released from neutrophils, vascular smooth muscle, macrophages, chondrocytes and cancer cells [18]. YKL-40 has been shown to induce activation of the mitogen-activated protein kinase pathway, nuclear factor-κB transcriptional activity and protein kinase B (Akt) pathway in cell cultures of human colon cell lines and human chondrocytes and synovial cells [9]. YKL-40 also potently stimulates the growth of several types of human fibroblasts derived from synovium, adult skin, and foetal lung [18]. Moreover, YKL-40 acts a chemoattractant that modulates vascular endothelial cell morphology by promoting the formation of branching tubules [19]. A recent study by Tang et al. demonstrated that YKL-40 may be involved in the inflammation of asthma by induction of IL-8 from epithelium, which subsequently contributes to BSMC proliferation and migration [20]. Collectively, YKL-40 has a role in inflammation, pathological, fibrosis and tissue remodelling.

Chupp et al. demonstrated that YKL-40 was highly unregulated in alveolar macrophages and the subepithelial basement membrane of asthmatic patients. Additionally, the serum YKL-40 level was elevated in asthmatic patients [10]. Duru et al. showed that serum YKL-40 levels were higher in non-smoker asthma patients during acute exacerbation than in control individuals [21]. Kuepper et al. demonstrated that YKL-40 levels were predominantly increased at the site of allergen deposition in response to allergen challenge [22]. A recent study suggested that serum YKL-40 levels were significantly elevated in patients with asthma compared to controls. This result indicates that high levels of serum YKL-40 may be a biological characteristic of asthma exacerbation [23].

Although previous studies have reported elevated YKL-40 levels in asthmatic patients, the change in serum YKL-40 levels upon treatment in asthmatic patients and its correlation with lung function and the asthma control test (ACT) remain unknown. Therefore, this study was conducted to determine whether serum YKL-40 levels are decreased in patients with asthma after the introduction of appropriate treatment. Furthermore, the relationships between the serum YKL-40 level, lung function and ACT were also investigated to evaluate the clinical significance of the serum YKL-40 level during the course of the disease.

## Methods

### Study subjects

This study randomly recruited 103 patients (47 women and 56 men, mean age 33.1 ± 0.9 years) from the Outpatient Clinic, Pulmonary Department, Affiliated Hospital of Guangdong Medical College. Patients with no previous diagnosis of asthma presenting with respiratory symptoms were considered eligible for our study when they were (a) older than 18 years of age, (b) able to perform spirometry, and (c) literate in Chinese. Asthma was diagnosed according to the GINA guidelines based on a history of recurrent episodes of wheezing and chest tightness with or without cough and impaired spirometry with reversibility in FEV_1_ of ≥12% and ≥200 ml after salbutamol administration or hyperresponsiveness to inhaled methacholine [24]. The exclusion criteria were the following: current smoking or smoking history of >5 pack years, oral corticosteroids or respiratory tract infection within the preceding 4 weeks prior to enrolment, any chronic cardiopulmonary disease other than asthma (including COPD) and pregnancy. We also obtained specimens from the Clinical Research Centre of Guangdong Medical College Tissue Bank. Although these samples were not specifically collected for this study, our study was part of a project that examined the molecular mechanism of inflammation in asthma. Bronchial-biopsy specimens were collected from 5 normal subjects who were non-smokers, 8 patients with mild asthma, 8 patients with moderate asthma and 7 patients with severe asthma. The basic characteristics matched those of the present study participants.

The study protocol was approved by the Ethics of Research Committee of the Medical College of Guangdong and was registered on the Chinese Clinical Trial Database (ChiCTR-OCC-13003316, http://www.chictr.org/en/proj/show.aspx?proj=5084). Written informed consent was obtained from all participants.

### Study design

At the baseline visit, all patients underwent a detailed clinical examination and completed a pulmonary function test, skin prick test (SPT), serum YKL-40 measurement, and ACT questionnaire. The patients were treated with appropriate medication according to the GINA guidelines [24] and were re-evaluated 8 weeks later (visit 2). The serum YKL-40 level, spirometry and ACT score were measured at the follow-up visit. Intermittent asthmatic patients received inhaled salbutamol 100-400 μg per day (Salbutamol Sulfate for Inhalation, GlaxoSmithKline) as needed. The mild to moderate and severe asthmatics were treated with low- to medium-dose budesonide (200-800 μg per day) delivered via turbuhalers (AstraZeneca AB) on a regular basis. These patients also received a beta-2 agonist (either a short-acting beta-2 agonist on an as-needed basis or a long-acting beta-2 agonist on a regular basis) and/or a third controller (e.g., leukotriene antagonists or aminophylline). The patient adherence to medical treatment was assessed using the Chinese version of the Medication Adherence Report Scale for Asthma (MARS-A10) items (Additional file 1: Table S1). MARS-A10 was developed by Horne and collaborators and is a brief self-measure with good psychometric properties [25].

### Pulmonary function tests

Spirometry was measured with standard spirometric techniques (Jaeger, Germany) at least 6 hours after a patient’s most recent treatment with albuterol, which is consistent with the American Thoracic Society (ATS) guidelines [26]. The patients were divided into the following three groups based on asthma severity according to GINA [24]: (1) mild asthma (FEV_1_ ≥ 80%); (2) moderate asthma (FEV_1_ 60-79%); and (3) severe asthma (FEV_1_ < 60%).

### Skin prick test (SPT) and asthma control test (ACT)

Atopy was tested with the SPT. Twelve common animal and aeroallergens (ALK-Abello, Horsholm, Denmark) were tested. The test was considered positive if the wheal diameter was at least 3 mm. The ACT questionnaires administered to the patients of this study had been formally translated into Chinese. Patients were classified into the following three groups based on ACT scores [27]: completely controlled (ACT score = 25), partly controlled (ACT score range = 20-24), and uncontrolled (ACT score range = 5-19). Asthma-related quality of life (QoL) was also measured using the Mini-Asthma QoL Questionnaire (Mini-AQLQ). The mini AQLQ is a validated 15-item questionnaire that assesses the quality of life specifically in relation to asthma [28]. The questionnaire assesses three domains of asthma interference with daily life (symptoms, environmental limitations, and emotions). The total score obtained in the different domains was divided by 15 to obtain the score for each patient. In this questionnaire, a higher score indicates a better quality of life [28].

### Serum YKL-40 and total serum IgE

The patient serum YKL-40 levels were measured using a commercially available enzyme-linked immunosorbent assay (ELISA) kit (Uscn Life Science Inc. Wuhan) according to the manufacturer’s instructions. The minimum detection limit of the YKL-40 assay was 12.2 pg/ml. The total serum IgE levels were determined using a fluoroenzyme immunoassay from asthmatic patients. The percentage of peripheral blood eosinophils was detected using a routine blood test.

### Immunohistochemistry

Deparaffinised sections (5-μm thick) were immersed in citrate antigen retrieval solution and preheated to 100°C for 20 minutes. The endogenous peroxidase was blocked with immunol staining blocking buffer for 60 min. The slides were then incubated with a rabbit polyclonal Ab against human YKL-40 (Uscn Life Science Inc., Wuhan, China). The immunoreaction was visualised using 3,3-diaminobenzidine chromogen solution (DAB substrate kit, Abcam, Cambridge, UK), and the sections were counterstained with hematoxylin according to the manufacturer’s instructions. Quantitative measurements of YKL-40-positive cells in the bronchial tissue were performed according to previously described methods [29]. Briefly, YKL-40-positive and -negative cells were counted in each biopsy specimen separately in the intact epithelium and the submucosa. The bronchial cells positive for the YKL-40 antibody staining were expressed as a percentage of total cells.

### Statistical analysis

The Kolmogorov-Smirnov (KS) test was performed to examine the normality of distribution. YKL-40 levels were not normally distributed, and the values were compared among the study groups using nonparametric tests including the Mann–Whitney U–test and the Kruskal–Wallis test. The interrelationships between different parameters were determined using Spearman’s correlation. The receiver operating characteristic (ROC) curve analysis was performed to derive the optimal cut-off value for YKL-40 as a predictor for an increase in FEV_1_ and response to steroid treatment. All data are presented as the mean ± SEM or n (%) unless otherwise stated. GraphPad Prism 5.0 software (GraphPad Software Inc., San Diego, CA, USA) was used for the analyses and graphs. Statistical significance was set at a *p-*value < 0.05.

## Results

### Patient characteristics

This study examined 103 patients with newly diagnosed asthma (56 men, 47 women, mean age 33.1 ± 0.9). There were 72 patients with mild asthma (intermittent or mild), 20 with moderate asthma and 11 with severe asthma. Sixty-nine patients (67.0%) had at least one positive SPT result. The most common sensitivities were to house dust mites and cockroaches. The mean baseline serum IgE, blood eosinophils in WBCs and body mass index (BMI) were 631.7 kU/L, 5.4% and 21.6, respectively. The characteristics of participants in our study are shown in Table 1.

**Table 1:** **Characteristic of the study participants**

Characteristic	Visit 1 (n = 103)	Visit 2 ^†^ (n = 98)	***p*** -value
Female, n (%)	47 (45.6)	45 (45.9)	NS
Age, yrs	33.1 ± 0.9	33.2 ± 0.9	NS
Body mass index	21.6 ± 0.3	21.4 ± 0.2	NS
Nonsmokers, n (%)	81 (78.6)	NA	
Ex-smokers, n (%)	22 (21.4)	NA	
Atopic by skin prick, n (%)	69 (67.0)	NA	
Total IgE, kU/L	631.7 ± 94.1	568.4 ± 86.8	NS
Eosinophil in WBC%	5.4 ± 0.4	4.0 ± 0.3	< 0.001
FEV_1_/FVC, %	72.1 ± 0.6	74.9 ± 0.8	0.0026
FEV_1_, % predicted	84.1 ± 1.4	87.8 ± 0.9	0.0270
≥80%, n (%)	72 (69.9)	85 (86.7)	
60-79%, n (%)	20 (19.4)	10 (10.2)	
< 60%, n (%)	11 (10.7)	3 (3.1)	
YKL-40, ng/ml	75.2 (55.8-86.8)	54.5 (46.4-58.4)	< 0.001
AQLQ	5.19 ± 0.11	5.35 ± 0.10	NS
ACT score	20.6 ± 0.5	22.1 ± 0.3	0.0101
25, n (%)	26 (25.2)	31 (31.6)	
20-24, n (%)	52 (50.5)	60 (61.3)	
5-19, n (%)	25 (24.3)	7 (7.1)	

Data are presented as mean ± SEM or n (%), except for YKL-40, median (IQR).

Note: FEV_1_, forced expiratory volume in one second; FVC, forced vital capacity; AQLQ, Asthma Quality of Life Questionnaire; ACT, asthma control test; NS, not significant; NA, not assessed.

^**†**^Five patients were dropped out.

During the follow-up visit, 5 patients were removed because they were unwilling to complete the treatment and refused to continue participating. Thus, 98 subjects completed the study and were included in the data analyses. There were no significant differences in baseline characteristics between patients who dropped out and patients who completed the treatment (Additional file 2: Table S2).

### Baseline visit

**Figure 1:**
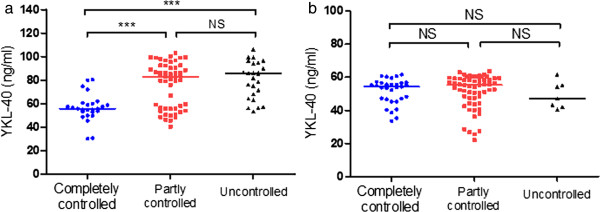
**Classification of patients based on ACT score at baseline visit (a) and visit 2 (b).** Note: ***p < 0.0001; NS: not significant. Horizontal bars indicate mean values.

At the baseline visit, the mean %FEV_1_ was 84.1 ± 1.4% and 72 patients (69.9%) had FEV_1_ ≥ 80%, 20 (19.4%) patients had FEV_1_ 60-79%, and 11 (10.7%) patients had FEV_1_ < 60% (Table 1). The mean ACT score was 20.6 ± 0.5, and 26 patients (25.2%) were completely controlled, 52 (50.5%) patients were partly controlled, and 25 (24.3%) patients were uncontrolled (Table 1). The patients with completely controlled asthma had statistically lower serum YKL-40 levels than patients with partly controlled (*p* < 0.001) and uncontrolled asthma (*p* < 0.001). However, there were no differences in the serum YKL-40 levels between the partially controlled group and the uncontrolled group (Figure 1a).

**Figure 2:**
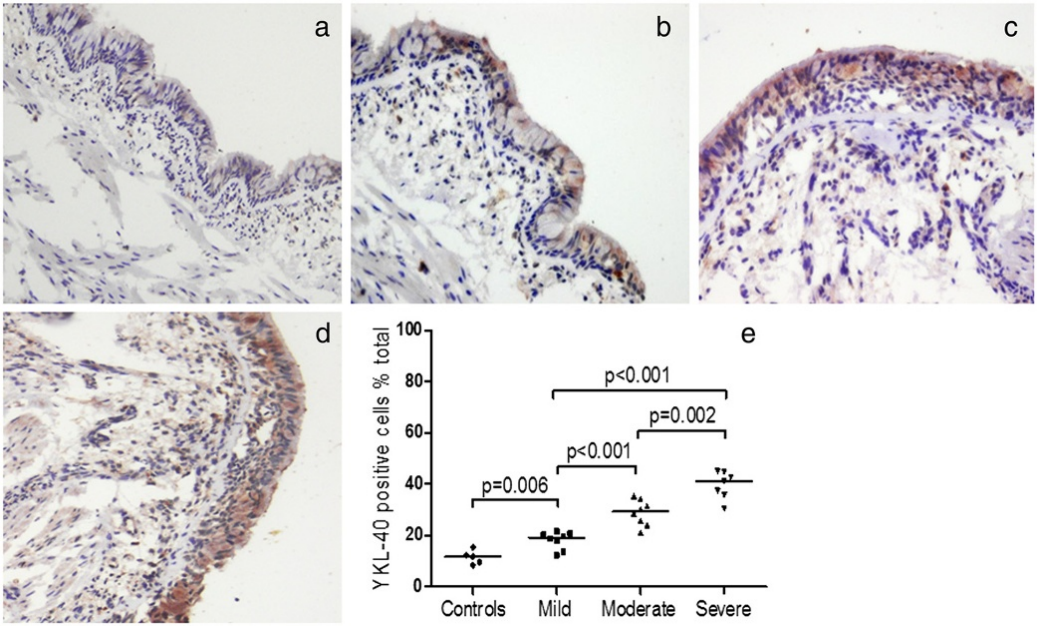
**YKL-40 is unregulated in the bronchial tissue of patients with asthma.** Specific staining for YKL-40 is brown, whereas nuclei are stained blue. Representative photomicrographs are present from **a)** a healthy individual, **b)** a patient with mild asthma, **c)** a patient with moderate asthma, and **d)** a patient with severe asthma. YKL-40-positive and -negative cells were counted in the epithelium and submucosa (expressed as a percentage of total cells). The data are presented as medians. **e)** YKL-40 is increased in both severe and moderate asthma compared with mild asthma, and there is a significant difference found between the two patient groups.

**Figure 3:**
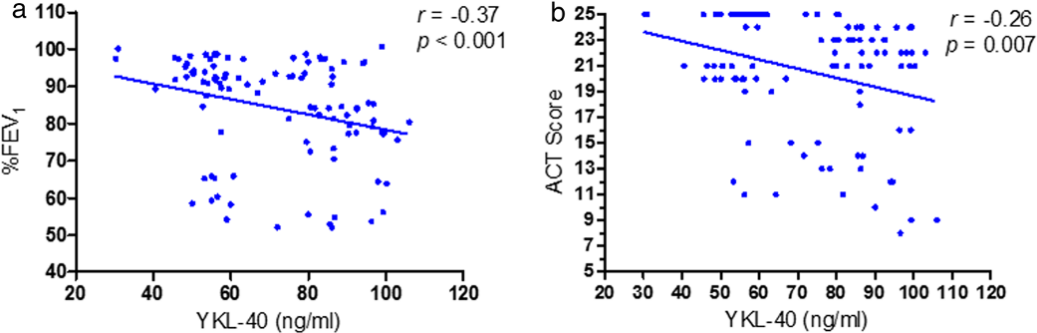
**Correlation of serum YKL-40 levels with FEV**_**1**_ **(a) and ACT score (b) at baseline visit.** Note: FEV_1_, forced expiratory volume in one second; ACT, asthma control test.

Serum YKL-40 levels in patients with severe asthma were higher than the levels in the moderate asthma group (85.60 [56.2-94.3] ng/ml vs. 76.00 [52.9-86.0] ng/ml; p = 0.024) and the mild asthma group (85.60 [56.2-94.3] ng/ml vs. 63.05 [53.6-75.3] ng/ml; p < 0.001). To confirm this result, bronchial biopsy specimens specifically stained for YKL-40 in patients with asthma were further evaluated. The quantification of lung YKL-40 expression revealed that YKL-40 expression was higher in mild asthma patients than in healthy controls (19.15% [14.60-20.75%] vs. 11.70% [9.01-13.85%]; p = 0.006). A subgroup analysis revealed that YKL-40 expression was higher in both severe and moderate asthma than mild asthma (41.0% [35.8-44.7%] vs. 19.2% [14.6-20.8%]; p < 0.001 and 29.2% [24.4-33.3%] vs. 19.2% [14.6-20.8%]; p < 0.001, respectively). Additionally, severe asthma exhibited significantly higher YKL-40 levels than mild asthma (41.0% [35.8-44.7%] vs. 29.2% [24.4-33.3%]; p = 0.002) (Figure 2). The serum YKL-40 level was negatively correlated with %FEV_1_ (*r* = -0.37, p < 0.001) and ACT score (*r* = -0.26, *p* = 0.007) at the baseline visit (Figure 3).

### Visit 2

**Figure 4:**
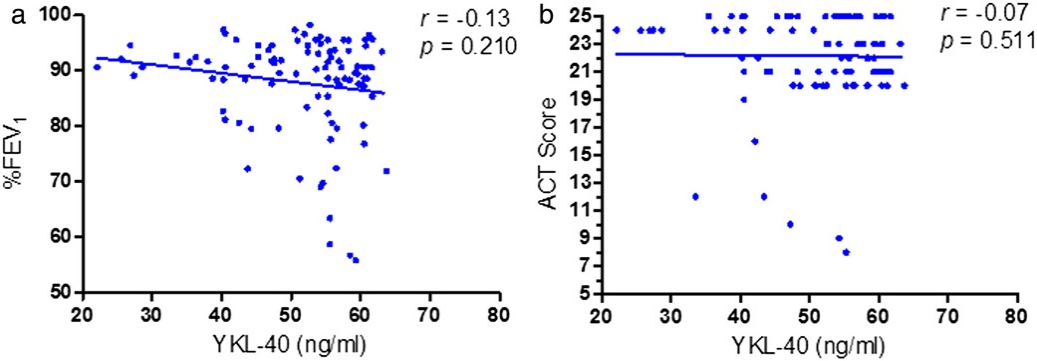
**Correlations of serum YKL-40 levels with (a) FEV**_**1**_ **and (b) ACT score at visit 2.** Note: FEV_1_, forced expiratory volume in one second; ACT, asthma control test.

At the follow-up visit, the median serum YKL-40 level was 54.5 [46.4-58.4] ng/ml and the mean %FEV_1_ was 87.8 ± 0.9%. There were 85 patients (86.7%) that had FEV_1_ ≥ 80%, 10 (10.2%) patients had FEV_1_ 60-79%, and 3 (3.1%) patients had FEV_1_ < 60% (Table 1). The mean ACT score was 22.1 ± 0.3. Thirty-one patients (31.6%) were completely controlled, 60 (61.3%) patients were partly controlled, and 7 (7.1%) patients had uncontrolled disease (Table 1). There were no statistically significant differences in serum YKL-40 values among patient groups with different ACT scores (Figure 1b). There were no correlations between serum YKL-40 and %FEV_1_ or ACT score values observed at visit 2 (*r* = -0.13, *p* = 0.210 and *r* = -0.07, *p* = 0.511, respectively) (Figure 4).

### Baseline visit versus visit 2

**Figure 5:**
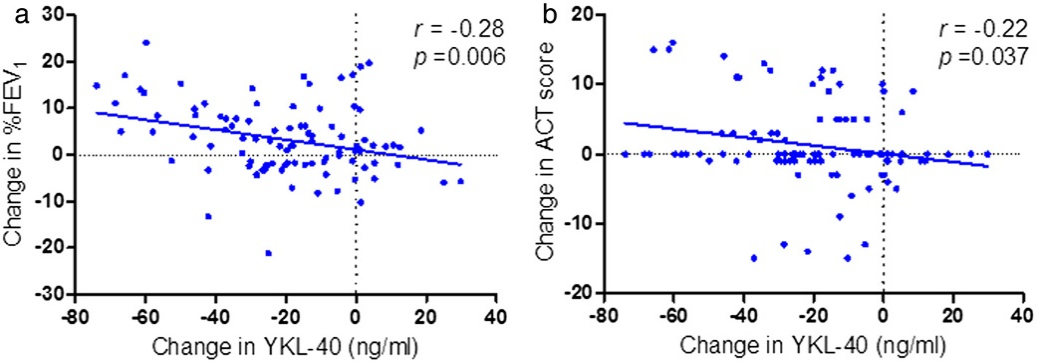
**Correlation of changes (Δ) in three parameters between the two visits.** Statistically significant correlations were observed **(a)** between ΔYKL-40 and ΔFEV_1_; **(b)** between ΔYKL-40 and ΔACT. Note: FEV_1_, forced expiratory volume in one second; ACT, asthma control test.

**Figure 6:**
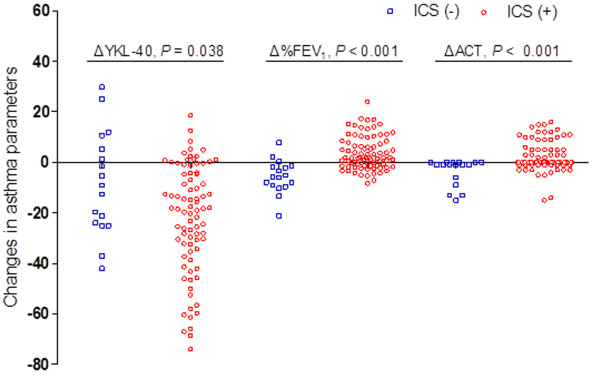
**Changes in asthma parameters in patient groups according to medication.** Note: FEV_1_, forced expiratory volume in one second; ACT, asthma control test. ICS, inhaled corticosteroids; ICS (+), patients with ICS treatment; ICS (-), patients without ICS treatment.

As shown in Table 1, there were statistically significant differences in serum YKL-40, %FEV_1_ value and ACT score between visit 1 and visit 2. We observed statistically significant correlations between changes (Δ) in YKL-40 and ΔFEV_1_ (*r* = -0.28, *p* =0.006) (Figure 5a) and between ΔYKL-40 and ΔACT (*r* = -0.22, *p* = 0.037) (Figure 5b). The patients who received ICS showed a decrease in serum YKL-40 levels and an improvement in %FEV_1_ and in the ACT score. The mean ΔYKL-40, Δ %FEV_1_ and ΔACT values differed significantly between the two medication groups (Figure 6). The outcomes and the relationship between treatment with ICS and response were assessed based on the low-dose administration of ICS (≤400 μg budesonide per day) and medium- to high-dose administration of ICS (>400 μg budesonide per day). Patients receiving low-dose ICS have lower YKL-40 levels and higher %FEV_1_ and ACT score at baseline compared to patients receiving medium- to high-dose ICS (Table 2). The patients with elevated levels of YKL-40 had significantly greater corticosteroid use than patients with lower levels (Table 2).

**Table 2:** **Efficacy results in asthma patients at baseline and after treatment with budesonide**

Variable	≤400 μg budesonide per day (n=50)		>400 μg budesonide per day (n=31)	
	Baseline	Visit 2	Baseline	Visit 2
FEV_1_, % predicted	90.8 (0.8)	91.2 (0.6)	71.6 (2.3)*	82.0 (1.8)^†^
ACT score	21.3 (0.7)	22.4 (0.5)	19.1 (0.6)^§^	22.5 (0.4)^†^
YKL-40, ng/ml	59.1 (54.3-86.6)	47.5 (40.3-55.3)^†^	86.2 (56.4-96.7)*	55.7 (43.8-63.7)^†^

Data are presented as mean ± SEM, except for YKL-40, median (IQR).

^†^p < 0.001 vs. baseline.

^§^p < 0.001 vs. baseline (≤400 μg budesonide per day).

*p < 0.01 vs. baseline (≤400 μg budesonide per day).

**Figure 7:**
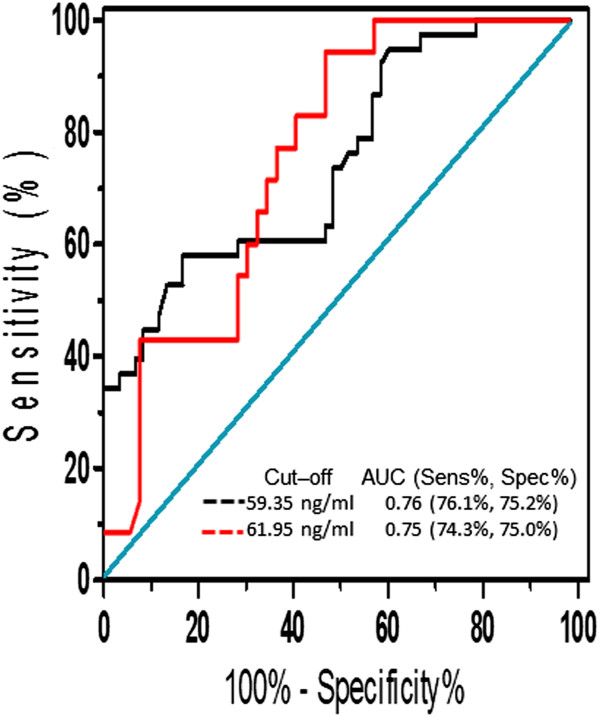
**Receiver operating characteristic curve showing YKL-40 as a predictor for an increase in FEV**_**1**_ **after treatment (black line) and as a predictor for response to steroid treatment (red line).** Sens, sensitivity; Spec, specificity; AUC, area under the ROC curve.

The values of the area under the ROC curve (AUC) for FEV_1_ and steroid treatment were 0.76 (95%CI 0.67 to 0.86, p < 0.001) and 0.75 (95%CI 0.63 to 0.85, p = 0.002), respectively (Figure 7).

The data indicate that serum YKL-40 levels were positively correlated with blood eosinophils (r = 0.29, p = 0.003) and were weakly positively correlated with total IgE levels (r = 0.18, p = 0.058). There were no correlations observed between serum YKL-40 levels, FEV_1_/FVC (%), age, SPT, BMI and Mini-AQLQ.

## Discussion

In this study, we explored the potential role of serum YKL-40 measurement in the management of asthma. There were several main findings of our study as follows: (i) elevated YKL-40 levels in asthma patients were associated with disease severity, and serum YKL-40 levels were deceased in asthmatic patients after administration of appropriate treatment; (ii) the serum YKL-40 level is negatively correlated with %FEV_1_ and ACT score before asthma treatment; (iii) although these correlations were no longer observed after treatment, changes in serum YKL-40, %FEV_1_ and ACT score continued to correlate significantly; and (iv) patients who received ICS showed a significant improvement in serum YKL-40, %FEV_1_ and ACT compared with patients without ICS. Patients with elevated levels of YKL-40 had significantly greater corticosteroid use than patients with lower levels. These results suggest that the YKL-40 level is implicated in the pathophysiology of asthma.

At baseline, the serum YKL-40 levels in the partly controlled group and uncontrolled group were significantly higher than the levels in the controlled group. However, there were no differences in the serum YKL-40 levels between the partly controlled group and the uncontrolled group. The reason for this finding is unclear, but it may be explained by the well-known individual variability in the perception of symptoms. The patients with uncontrolled ACT (≤19)/YLK-40 levels below the median (≤75.2) (Additional file 3: Figure S1A) may have adequately addressed the atopic inflammation in their airways after treatment. However, these patients continue to have symptom persistence for other reasons including co-morbidities (e.g., rhinitis, gastroesophageal reflux disease), non-eosinophilic asthma and other conditions that may be similar to asthma. The patients who had ACT controlled (>19)/YLK-40 levels above the median (>75.2) (Additional file 3: Figure S1B) were symptomatically controlled but have evidence of persistent inflammation and could be at risk for future asthma problems. Moreover, those who were ACT uncontrolled (≤19)/YLK-40 levels above the median (>75.2) (Additional file 3: Figure S1C) may be part of the symptom controlled/high level of YKL-40 (>75.2) asthmatics group. This result could be partly explained by a poor symptom-perception due to adaptation to their level of disease. These data were consistent with previous reports that suggested that asthma evaluation cannot be inferred from single measures of ACT score [29–32]. GINA also recommends that questionnaires addressing the level of asthma control need to be considered in combination with objective measurements such as lung function and inflammatory cytokines [24]. To reconcile this issue, we further performed a separate analysis and reanalysed the data using the asthma severity according to GINA. The data showed that serum YKL-40 levels for patients in the severe asthma group were higher than the levels in the moderate and mild asthma groups. To confirm this result, bronchial biopsy specimens were specifically stained for YKL-40 in patients with asthma and were then further evaluated. The quantification of lung YKL-40 expression revealed that YKL-40 expression was higher in mild asthma patients than healthy controls. The subgroup analysis revealed that severe asthma patients have significantly higher YKL-40 levels than mild and moderate asthma patients. These findings suggest that the ACT questionnaire may convey something that inflammatory markers and spirometry cannot assess. However, asthma evaluation control cannot be inferred from single measures of ACT score and should be considered with objective measurements (e.g., biomarkers, lung function). The YKL-40 level is upregulated in human asthma and is associated with disease severity, which suggests that high levels of YKL-40 may be a biological characteristic of asthma severity.

We observed statistically significant correlations between the serum YKL-40 level and %FEV_1_ and between the serum YKL-40 level and ACT score. Specjalski et al. showed that in a Polish population, the serum levels of YKL-40 were significantly higher in patients with uncontrolled and partly controlled asthma than in controlled asthmatic patients and healthy subjects [33]. Chupp et al. demonstrated that the circulating YKL-40 level was correlated with lung function, asthma severity, and the thickness of the subepithelial basement membrane [10]. Additionally, a recent study by Tang et al. showed that serum YKL-40 levels correlated positively with exacerbation attacks and blood eosinophils. However, the levels were correlated inversely with lung function [23]. The exact role of the serum YKL-40 level in asthma is still unclear and controversial [33–35]. Specjalski et al. showed that there were no relationships between YKL-40 and asthma severity or total serum IgE [33]. A recent study by Sohn et al. suggested that there were no significant associations between CHI3L1 single nucleotide polymorphisms (SNPs) and asthma in an East Asian population [34]. The basis for these discrepancies may be due to ethnic background and differences in CHI3L1 haplotype structures. The CHI3L1 polymorphisms have been associated with features of asthma and serum YKL-40 levels [36–38]. This suggests that the CHI3LI gene polymorphism may lead to a reduction in YKL-40.

At visit 2, no correlations between serum YKL-40 and %FEV_1_ or ACT score were observed. One explanation may be that a majority of patients in our study were mild asthmatics and showed a response in all three measurements after administration of appropriate therapy, which minimised the range of their distribution. However, the changes in serum YKL-40, %FEV_1_ and ACT score were significantly correlated. Furthermore, the serum YKL-40 level declined in asthmatic patients after appropriate treatment. One possible explanation for this result could be that ACT scores were significantly improved in most patients who presented with a mild or stable condition after treatment. This result is consistent with previous studies by Chupp et al. and Tang et al. that showed that serum YKL-40 levels were higher in patients with exacerbated asthma than in stable patients [10]. In addition, our data demonstrated that patients with asthma receiving regular ICS therapy had a significant improvement in serum YKL-40, %FEV_1_, and ACT. Patients with elevated levels of YKL-40 had significantly greater corticosteroid use than patients with lower levels. This result suggests that YKL-40 production may be refractory to current asthma treatments and may represent an alternative therapeutic target for severe asthma [39]. A previous study demonstrated that serum YKL-40 levels in patients with active rheumatoid arthritis decreased rapidly during prednisolone therapy, which suggests that steroids may have a direct effect on expression of YKL-40 [40]. However, it should be noted that patients were treated with ICS, but differences in dosing and compliance existed and serum YKL-40 levels may be influenced by the CHI3LI gene polymorphism or other medications such as bronchodilators.

There are some limitations to this study that need to be considered. Asthmatic patients with newly diagnosis asthma were enrolled in our study, and most patients (69.9%, 72/103) had mild asthma. Our results may not fully reflect the general population of asthmatic patients, especially patients with severe therapy-resistant asthma. Additionally, we did not consider the Asthma Control Questionnaire (ACQ) because the ACQ has not been validated in the Chinese population and the ACT is easier to score and provides the same screening accuracy [29, 41, 42].

## Conclusions

In summary, our current findings demonstrate that elevated YKL-40 levels in asthma patients correlate with disease severity. Patients with elevated levels of YKL-40 had significantly greater corticosteroid use than patients with lower levels, which suggests that high levels of serum YKL-40 may be refractory to current asthma treatments. Future prospective studies are required to determine the role of YKL-40 in patients with severe therapy-resistant asthma and will assess whether YKL-40 gene expression is directly controlled by corticosteroids.

## Electronic supplementary material

Additional file 1: Table S1: The Medication Adherence Report Scale for Asthma (MARS-A10). (DOCX )

Additional file 2: Table S2: Characteristics of the study participants and participants excluded. (DOCX )

Additional file 3: Figure S1: Correlation of serum YKL-40 levels with ACT score at baseline visit. A: ACT remain uncontrolled (≤19)/YLK-40 levels below the median (≤75.2); B: ACT controlled (>19)/YLK-40 levels above the median (>75.2); C: ACT uncontrolled (≤19)/YLK-40 levels above the median (>75.2). (TIF )

## Abbreviations

ACT: Asthma control test
ICS: Inhaled corticosteroids
CHI3L1: Chitinase 3-like 1
COPD: Chronic obstructive pulmonary disease
GINA: Global initiative for asthma
SPT: Skin prick test
LABA: Long acting β_2_-agonist
SABA: Short acting β_2_-agonist
ATS: American thoracic society
FEV_1_: Forced expiratory volume in one second
FVC: Forced vital capacity
AQLQ: Asthma QoL Questionnaire.

## References

[1] Wark PA, Murphy V, Mattes J (2014). The interaction between mother and fetus and the development of allergic asthma. Expert Rev Respir Med.

[2] Collison A, Li J, Pereira de Siqueira A, Zhang J, Toop HD, Morris JC (2014). Tumor necrosis factor-related apoptosis-inducing ligand regulates hallmark features of airways remodeling in allergic airways disease. Am J Respir Cell Mol Biol.

[3] Gaga M, Papageorgiou N, Zervas E, Gioulekas D, Konstantopoulos S (2005). Control of asthma under specialist care: is it achieved?. Chest.

[4] Boot GR, van Achterberg AE, van Aken BE, Renkema GH, Jacobs MJHM, Aerts JM (1999). Strong induction of members of the chitinase family of proteins in atherosclerosis. Arterioscler Thromb Vasc Biol.

[5] Johansen JS, Jensen BV, Roslind A, Nielsen D, Price PA (2006). Serum YKL-40, a new prognostic biomarker in cancer patients?. Cancer Epidem Biomarkers Prev.

[6] Johansen JS, Chrisoffersen P, Moller S, Price PA, Henriksen JH, Garbarsch C (2006). Serum YKL-40 is increased in patients with hepatic fibrosis. J Hepatol.

[7] Kucur M, Isman FK, Karadag B, Vural VA, Tavsanoglu S (2007). Serum YKL-40 levels in patients with coronary artery disease. Coron Artery Dis.

[8] Kim SH, Das K, Noreen S, Coffman F, Hameed M (2007). Prognostic implicationsof immunohistochemically detected YKL-40 expression in breast cancer. World J Surg Oncol.

[9] Rathcke CN, Johansen JS, Vestergaard H (2006). YKL-40, a biomarker of inflammation, is elevated in patients with type 2 diabetes and is related to insulin resistance. Inflamm Res.

[10] Chupp GL, Lee CG, Jarjour N, Shim YM, Holm CT, He S (2007). A chitinase-like protein in the lung and circulation of patients with severe asthma. N Engl J Med.

[11] Furuhashi K, Suda T, Nakamura Y, Inui N, Hashimoto D, Miwa S (2010). Increased expression of YKL-40, a chitinase-like protein, in serum and lung of patients with idiopathic pulmonary fibrosis. Respir Med.

[12] Létuvé S, Kozhich A, Arouche N, Grandsaigne M, Reed J, Dombret MC (2008). YKL-40 is elevated in patients with chronic obstructive pulmonary disease and activates alveolar macrophages. J Immunol.

[13] Fantino E, Gangell CL, Hartl D, Sly PD, AREST CF (2014). Airway, but not serum or urinary, levels of YKL-40 reflect inflammation in early cystic fibrosis lung disease. BMC Pulm Med.

[14] Holmgaard DB, Mygind LH, Titlestad IL, Madsen H, Pedersen SS, Johansen JS (2013). Plasma YKL-40 and all-cause mortality in patients with chronic obstructive pulmonary disease. BMC Pulm Med.

[15] Ortega H, Prazma C, Suruki RY, Li H, Anderson WH (2013). Association of CHI3L1 in African-Americans with prior history of asthma exacerbations and stress. J Asthma.

[16] Guerra S, Halonen M, Sherrill DL, Venker C, Spangenberg A, Carsin AE (2013). The relation of circulating YKL-40 to levels and decline of lung function in adult life. Respir Med.

[17] Saba M, Sharif MR, Akbari H, Nikoueinejad H, Ramazani Jolfaii M (2014). YKL-40 in Asthma and its correlation with different clinical parameters. Iran J Allergy Asthma Immunol.

[18] Zhu Z, Zheng T, Homer RJ, Kim YK, Chen NY, Cohn L (2004). Acidic mammalian chitinase in asthmatic Th2 inflammation and IL-13 pathway activation. Science.

[19] Malinda KM, Ponce L, Kleinman HK, Shackelton LM, Millis AJ (1999). Gp38k, a protein synthesized by vascular smooth muscle cells, stimulates directional migration of human umbilical vein endothelial cells. Exp Cell Res.

[20] Tang H, Sun Y, Shi Z, Huang H, Fang Z, Chen J (2013). YKL-40 induces IL-8 expression from bronchial epithelium via MAPK (JNK and ERK) and NF-κB pathways, causing bronchial smooth muscle proliferation and migration. J Immunol.

[21] Duru S, Yüce G, Ulasli SS, Erdem M, Kizilgün M, Kara F (2013). The relationship between serum YKL-40 levels and severity of asthma. Iran J Allergy Asthma Immunol.

[22] Kuepper M, Bratke K, Virchow JC (2008). Chitinase-like protein and asthma. N Engl J Med.

[23] Tang H, Fang Z, Sun Y, Li B, Shi Z, Chen J (2010). YKL-40 in asthmatic patients, and its correlations with exacerbation, eosinophils and immunoglobulin E. Eur Respir J.

[24] Global Initiative for Asthma: *GINA Report: Global strategy for asthma management and prevention (2011 Update)*. (accessed 20 December 2012) http://www.ginasthma.com

[25] Mora PA, Berkowitz A, Contrada RJ, Wisnivesky J, Horne R, Leventhal H (2011). Factor structure and longitudinal invariance of the Medical Adherence Report Scale-Asthma. Psychol Health.

[26] American Thoracic Society (1995). Standardization of spirometry (1994 update). Am J Respir Crit Care Med.

[27] Nathan RA, Sorkness CA, Kosinski M, Schatz M, Li JT, Marcus P (2004). Development of the asthma control test: a survey for assessing asthma control. J Allergy Clin Immunol.

[28] Juniper EF, Guyatt GH, Cox FM, Ferrie PJ, King DR (1999). Development and validation of the Mini Asthma Quality of Life Questionnaire. Eur Respir J.

[29] Xanthou G, Alissafi T, Semitekolou M, Simoes DC, Economidou E, Gaga M (2007). Osteopontin has a crucial role in allergic airway disease through regulation of dendritic cell subsets. Nat Med.

[30] Jia CE, Zhang HP, Lv Y, Liang R, Jiang YQ, Powell H (2013). The Asthma Control Test and Asthma Control Questionnaire for assessing asthma control: Systematic review and meta-analysis. J Allergy Clin Immunol.

[31] Piacentini GL, Peroni DG, Bodini A, Bonafiglia E, Rigotti E, Baraldi E (2009). Childhood Asthma Control Test and airway inflammation evaluation in asthmatic children. Allergy.

[32] Senna G, Passalacqua G, Schiappoli M, Lombardi C, Wilcock L (2007). Correlation among FEV1, nitric oxide and asthma control test in newly diagnosed asthma. Allergy.

[33] Specjalski K, Jassem E (2011). YKL-40 protein is a marker of asthma. J Asthma.

[34] Sohn MH, Lee JH, Kim KW, Kim SW, Lee SH, Kim KE (2009). Genetic variation in the promoter region of chitinase 3-like 1 is associated with atopy. Am J Respir Crit Care Med.

[35] Santos CB, Davidson J, Covar RA, Spahn JD (2014). The chitinase-like protein YKL-40 is not a useful biomarker for severe persistent asthma in children. Ann Allergy Asthma Immunol.

[36] Ober C, Tan Z, Sun Y, Possick JD, Pan L, Nicolae R (2008). Effect of variation in CHI3L1 on serum YKL-40 level, risk of asthma, and lung function. N Engl J Med.

[37] Konradsen JR, James A, Nordlund B, Reinius LE, Söderhäll C, Melén E (2013). The chitinase-like protein YKL-40: a possible biomarker of inflammation and airway remodeling in severe pediatric asthma. J Allergy Clin Immunol.

[38] Tsai Y, Ko Y, Huang M, Lin M, Wu C, Wang C (2014). CHI3L1 polymorphisms associate with asthma in a Taiwanese population. BMC Med Genet.

[39] Hartl D, Lee CG, Da Silva CA, Chupp GL, Elias JA (2009). Novel biomarkers in asthma: chemokines and chitinase-like proteins. Curr Opin Allergy Clin Immunol.

[40] Johansen JS, Stoltenberg M, Hansen M, Florescu A, Hørslev-Petersen K, Lorenzen I (1999). Serum YKL-40 concentrations in patients with rheumatoid arthritis: relation to disease activity. Rheumatology.

[41] Zhou X, Ding FM, Lin JT, Yin KS (2009). Validity of asthma control test for asthma control assessment in Chinese primary care settings. Chest.

[42] Zhou X, Ding FM, Lin JT, Yin KS, Chen P, He QY (2007). Validity of Asthma Control Test in Chinese patients. Chin Med J (Engl).

[CR43] The pre-publication history for this paper can be accessed here: http://www.biomedcentral.com/1471-2466/15/1/prepub

