# Supplementary material for: YKL-40 is correlated with FEV1 and the asthma control test (ACT) in asthmatic patients: influence of treatment
Source: BMC Pulm Med. 2015 Jan 12;15:1. doi: 10.1186/1471-2466-15-1 (PMC4417200; doi:10.1186/1471-2466-15-1)
Supplement: Supplementary file 1 — Additional file 1: Table S1: The Medication Adherence Report Scale for Asthma (MARS-A10). (DOCX ) [file 12890_2013_665_MOESM1_ESM.docx]

| **Table S1 The Medication Adherence Report Scale for Asthma (MARS-A10)** | |
| --- | --- |
| Item |  |
| 1. I only use my [Name of Medicine] when I need it | |
| 2. I only use it when I feel breathless | |
| 3. I decide to miss out a dose | |
| 4. I try to avoid using it | |
| 5. I forget to take it | |
| 6. I alter the dose | |
| 7. I stop taking it for a while | |
| 8. I use it as a reserve, if my other treatment doesn’t work | |
| 9. I use it before doing something which might make me breathless | |
| 10. I take it less than instructed | |
