# Supplementary material for: YKL-40 is correlated with FEV1 and the asthma control test (ACT) in asthmatic patients: influence of treatment
Source: BMC Pulm Med. 2015 Jan 12;15:1. doi: 10.1186/1471-2466-15-1 (PMC4417200; doi:10.1186/1471-2466-15-1)
Supplement: Supplementary file 2 — Additional file 2: Table S2: Characteristics of the study participants and participants excluded. (DOCX ) [file 12890_2013_665_MOESM2_ESM.docx]

| **Table S2 Characteristics of the study participants and participants excluded*** | | | |
| --- | --- | --- | --- |
| Characteristic | Study participants  (n = 98) | Participants excluded (n = 5) | *P*-value |
| Female, n (%) | 45 (45.9) | 2 (40.0) | 0.63 |
| Age, yrs | 32.7 ± 0.8 | 34.1 ± 0.3 | 0.69 |
| BMI | 21.8 ± 0.3 | 21.5 ± 0.2 | 0.82 |
| Total IgE, kU/L | 633.5 ± 93.6 | 618.3 ± 36.1 | 0.97 |
| Eosinophil in WBC% | 5.3 ± 0.4 | 5.0 ± 0.2 | 0.86 |
| FEV_1_/FVC, % | 71.6 ± 0.6 | 72.8 ± 0.3 | 0.65 |
| FEV_1_, % predicted | 84.6 ± 1.4 | 83.9 ± 0.7 | 0.91 |
| YKL-40, ng/ml | 75.7 (56.1-85.3) | 74.6 (52.4-83.7) | 0.60 |
| ACT score | 20.1 ± 0.5 | 20.9 ± 0.2 | 0.72 |
| AQLQ | 5.11 ± 0.11 | 5.41 ± 0.09 | 0.54 |
| *****Data are presented as mean ± SEM or n (%), except for YKL-40, median (IQR).  Note: BMI, Body mass index; FEV_1_, forced expiratory volume in one second; FVC, forced vital capacity; ACT, asthma control test; AQLQ, Asthma Quality of Life Questionnaire. | | | |
