# Supplementary figures and images for: YKL-40 is correlated with FEV1 and the asthma control test (ACT) in asthmatic patients: influence of treatment
Source: BMC Pulm Med. 2015 Jan 12;15:1. doi: 10.1186/1471-2466-15-1 (PMC4417200; doi:10.1186/1471-2466-15-1)

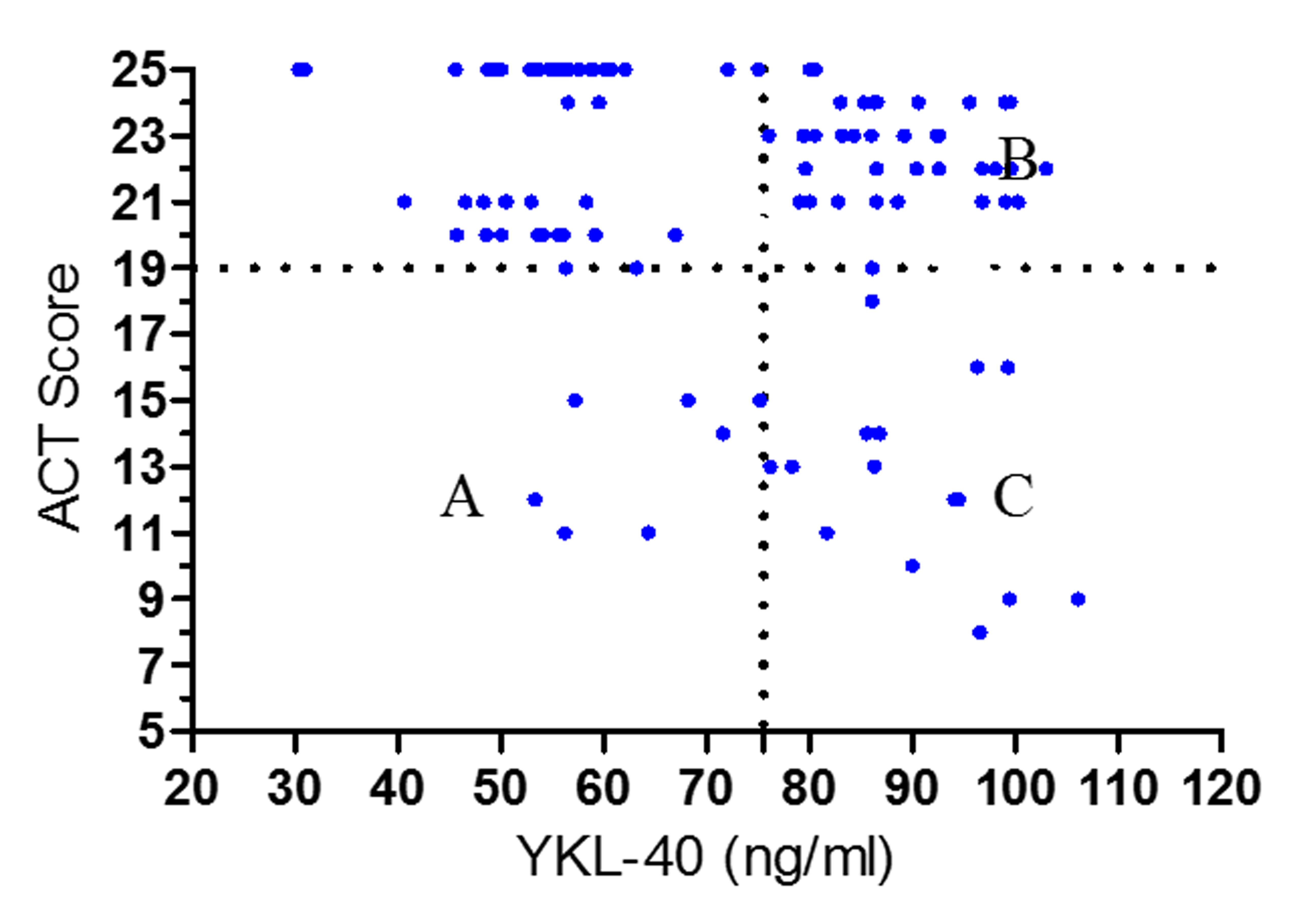

Supplement: Supplementary file 3 — Additional file 3: Figure S1: Correlation of serum YKL-40 levels with ACT score at baseline visit. A: ACT remain uncontrolled (≤19)/YLK-40 levels below the median (≤75.2); B: ACT controlled (>19)/YLK-40 levels above the median (>75.2); C: ACT uncontrolled (≤19)/YLK-40 levels above the median (>75.2). (TIF ) [file 12890_2013_665_MOESM3_ESM.tif]
